# The process of feature binding

**DOI:** 10.3389/fpsyg.2013.00207

**Published:** 2013-04-18

**Authors:** Snehlata Jaswal

**Affiliations:** Psychology, Cognitive Science, Department of Humanities and Social Sciences, Indian Institute of Technology DelhiNew Delhi, India

The integration of different stimulus properties as an object is the process of feature binding. The research topic “Inhibition in the process of feature binding” aimed to debate which features bind together, why, and how; and what happens to features which are not integrated.

The authors of the review articles took up the debate in earnest. Wyatte et al. (2012) emphasize that the process of binding does not require any special neural substrates. The two mechanisms of inhibition and top-down feedback are sufficient to explain the process of object recognition. Their computational model describes how these established principles of neural processing work over time to solve the binding problem. Particularly critical of the idea of neural synchrony, often held forth as “the” mechanism underlying feature binding, they contend that it helps only to create a contrast between relevant and irrelevant features. It is top-down feedback, which reinforces relevant information.

The relevance of features is the key factor in the process of binding for Jaswal (2012). She attempts an integration of physiological and psychological literature, highlighting that only relevant features bind together in a rather slow process, which concomitantly ensures that the irrelevant features are actively inhibited. In a more expanded overview, Velik (2012) reaches similar conclusions—that irrelevant information is deleted from the system in the process of binding. However, whilst Jaswal (2012) bases her conclusions on empirical studies, Velik (2012) presents a computational model in which filter mechanisms work to suppress irrelevant information. Krummenacher and Müller (2012) review evidence from behavioral and ERP studies to confirm that separate feature dimensions are important in the pre-selective phase of feature binding. However, once the bound object emerges, feature based effects are not as dominant. This corroborates with the aforementioned views that only relevant features are carried forward.

Herzog et al. (2012) also address how we process features prior to and during the process of binding. Using the sequential metacontrast paradigm, they explore binding as a sub-process in the Gestalt experience of grouping elements into wholes. They contend that computation of features is in itself a process and not an instantaneous event. Stimulus representations are dynamic and binding probably occurs simultaneously with the processing of independent features.

Meier and Rey-Mermet (2012) discuss episodic context binding using bivalent stimuli. The ambiguity of bivalent stimuli is itself a “feature” that enters binding and influences subsequent behavior in consonance with the bivalency effect. It is interesting that Meier and Ray-Mermet propose that this conflict is not relevant to the task. In fact, this ambiguity and concomitant conflict is a cardinal feature of bivalent stimuli, which reactivates whenever the context is redintegrated.

Moving from features to objects, Dent et al. (2012) review behavioral and physiological evidence for the process of distracter inhibition in visual search. They postulate that it is a resource demanding active process, which is parallel in nature, such that all distracters are deleted, and the target alone remains as the item to be processed further.

The four articles contributing original research extend the study of binding to novel avenues. As merits empirical investigations, all studies not only document details of current work in these new areas, they also indicate hypotheses for future investigations.

Delogu et al. (2012) enter the arena of audition to investigate the link between location and temporal order. The recall of temporal order is weakened more than locations, whilst binding in visual as well as auditory domains. This suggests that location is encoded relatively automatically during binding, but recall of temporal order is resource demanding. Similar results across visual and auditory domains suggest that binding of location and temporal order may involve shared, modality-independent physiological mechanisms. This special link between “when” and “where” features, merits future explorations.

Giersch et al. (2012) scale up from features to explore grouping and regrouping between objects among schizophrenics and healthy controls. Patients were particularly slow to detect connected targets when the attentional focus was on unconnected pairs. This effect was found only for targets presented within the same hemifield. Thus, schizophrenics do not regroup stimuli in the same way as healthy controls. Speculations can be made regarding the role of connectivity between hemispheres in grouping. Further, grouping and regrouping are different (maybe parallel) mechanisms, the former relying on automatic processing, whilst the latter demand attentional resources.

The two original research articles based on computational models conceptualize binding as part of other processes. Schrobsdorff et al. (2012) propose that binding is an essential phase in inhibition. In their model of the negative priming effect, features are activated, bound into object entities, and related to their context. Inhibitory processes and changing thresholds implement the concept of selective attention. Thus, binding becomes a sub-process in a general model of inhibition. Davelaar (2013) evokes the concept of binding to explain the Gratton effect in the Eriksen flanker task. The Gratton effect is the reduction in the interference due to flankers after incongruent, as compared with congruent trials. Results of experiments that separate the contributions from target, flanker, and response repetition, show that flanker repetition alone is sufficient to produce congruency effects in sequences of trials. He postulates that representations of targets, flankers, and response(s) are associated in a task set. This work is an excellent example of how the concept of binding is used to understand other ideas and paradigms.

The aim of this research topic was to consider the process of binding from various perspectives such that an integrated view emerged to guide future research and theory. Challenging the assumption that feature binding is an automatic “event” driven by bottom up processes dependent on conjunctively coding neurons or synchrony, the collection of these articles yield the conclusion that the emergence of a bound object capable of further processing, is itself a sub-process, which is heavily contingent on re-entrant mechanisms and is probably resource demanding. Future investigations may explore feature binding as a basic process in myriad behavioral sequences of diverse populations.
